# Characterisation of *Campylobacter jejuni *genes potentially involved in phosphonate degradation

**DOI:** 10.1186/1757-4749-1-13

**Published:** 2009-06-25

**Authors:** Lauren E Hartley, Nadeem O Kaakoush, Justin L Ford, Victoria Korolik, George L Mendz

**Affiliations:** 1 Institute for Glycomics, Griffith University, Gold Coast, Australia; 2 School of Medicine, Sydney, The University of Notre Dame Australia, Darlinghurst NSW 2010, Australia

## Abstract

Potential biological roles of the *Campylobacter jejuni *genes *cj0641, cj0774c *and *cj1663 *were investigated. The proteins encoded by these genes showed sequence similarities to the phosphonate utilisation PhnH, K and L gene products of *Escherichia coli*. The genes *cj0641, cj0774c *and *cj1663 *were amplified from the pathogenic *C. jejuni *strain 81116, sequenced, and cloned into pGEM-T Easy vectors. Recombinant plasmids were used to disrupt each one of the genes by inserting a kanamycin resistance (*Km*^R^) cassette employing site-directed mutagenesis or inverse PCR. *Campylobacter jejuni *81116 isogenic mutants were generated by integration of the mutated genes into the genome of the wild-type strain. The *C. jejuni *mutants grew on primary isolation plates, but they could not be purified by subsequent passages owing to cell death. The mutant *C. jejuni *strains survived and proliferated in co-cultures with wild-type bacteria or in media in which wild-type *C. jejuni *had been previously grown. PCR analyses of mixed wild-type/mutant cultures served to verify the presence of the mutated gene in the genome of a fraction of the total bacterial population. The data suggested that each mutation inactivated a gene essential for survival. Rates of phosphonate catabolism in lysates of *E. coli *strain DH5α were determined using proton nuclear magnetic resonance spectroscopy. Whole-cell lysates of the wild-type degraded phosphonoacetate, phenylphosphonate and aminomethylphosphonate. Significant differences in the rates of phosphonate degradation were observed between lysates of wild-type *E. coli*, and of bacteria transformed with each one of the vectors carrying one of the *C. jejuni *genes, suggesting that these genes were involved in phosphonate catabolism.

## Background

Phosphorus is an essential element for living organisms. In bacteria it has roles in biosynthesis and energy metabolism, as well as in the structure of biomolecules. Phosphonates (Phn) are a class of organophosphates structurally similar to phosphate esters, but characterised by a carbon-to-phosphorus (C-P) bond [1-3]. The C-P bond is highly stable compared to oxygen-to-phosphorus, nitrogen-to-phosphorus or sulphur-to-phosphorus bonds. The C-P bond is resistant to chemical hydrolysis, thermal decomposition, photolysis [4], and to the action of phosphatases [5,6]. During phosphate limitation stress, some bacteria can utilise phosphonates as an alternative source of phosphorus by breaking the C-P bonds of these compounds [7-9].

Four routes of phosphonate catabolism are known in microorganisms, namely, the phosphonoacetaldehyde hydrolase (phosphonatase), phosphonoacetate hydrolase, phosphonopyruvate hydrolase and C-P lyase pathways [6,10]. Cleavage mechanisms and substrate specificity are different for each pathway. C-P lyase pathways are able to cleave a variety of phosphonates including aminomethylphosphonate (AmePhn) and phenylphosphonate (PhePhn), the latter is not degraded by other enzymes of phosphonate catabolism [1,11-13].

Genes encoding C-P lyase pathways have been found in bacteria of the genera *Enterobacter, Escherichia, Klebsiella *and *Kluyvera *[1,11-14]; and phosphonatase pathways are found in the genera *Enterobacter *and *Salmonella *[11,15]. Recently, it was demonstrated that *Campylobacter *spp. are capable of degrading some phosphonate-bearing compounds although *in silico *analyses indicated that no genes orthologous to those encoding C-P bond-cleaving enzymes in other bacteria are present in the genome of *Campylobacter jejuni *[12]. Two different C-P bond cleavage activities were discovered in *Campylobacter *spp. The first one is associated with the cell wall and has the ability to catalyse phenylphosphonate and phenylphosphinate (PhePhi). The second cleavage activity is found in both the cell wall and cytosolic fractions and catalyses phosphonoalkyl carboxylates [12]. Phosphonate metabolism in *Campylobacter *does not require phosphate starvation conditions, unlike other bacteria in which the expression of Phn-degrading enzymes are under control of the Pho regulon [7,13,16].

The molecular basis of Phn catabolism by *C. jejuni *wasinvestigated in this study. *In silico *analyses were performed to identify *C. jejuni *genes encoding proteins with similarity to those involved in C-P bond hydrolysis in *E. coli *K12 [8]. Isogenic mutants of wild-type *C. jejuni *strain 81116 were constructed by disrupting with a kanamycin cassette three genes encoding proteins potentially implicated in phosphonate degradation, and the viability of the mutants was determined. Mutants of *E. coli *DH5α were generated by transforming the wild-type strain with plasmids carrying each one of the *C. jejuni *genes putatively involved in Phn catabolism. The rates of degradation of three phosphonate compounds by the mutant *E. coli *were compared to those of the wild-type.

## Results

### *In silico *analyses of genes putatively involved in phosphonate catabolism

Three genes orthologous to those involved in C-P bond cleavage in *E. coli *were identified in the genome of *C. jejuni *strain NCTC 11168 by protein homology analyses. The proteins encoded by the *C. jejuni *genes, *cj0774c *and *cj1663 *showed homology to the *E. coli *membrane-associated C-P lyase proteins PhnK and PhnL encoded by the *phnC-P *operon (Table 1). Domain architecture searches demonstrated that the four proteins contained the ABC_MJ0796 domain characteristic of some ATP-binding cassettes of ABC transport systems. The protein encoded by *cj0641 *showed low similarity to *E. coli *PhnH, but had 48% similarity to the inorganic polyphosphate/ATP-NAD kinase JHP1433 of *H. pylori *strain J99 an enzyme which utilizes ATP and other nucleoside triphosphates as well as inorganic polyphosphate as a source of phosphorus, and contains a COG3221.2 domain of the periplasmic component of ABC-type/phosphonate transport system. In comparison, the similarities of *E. coli *PhnH, PhnK and PhnL with proteins of the C-P lyase pathway of *Enterobacter sp. 638 *were 92, 98 and 92%, respectively; and the similarities with *Klebsiella pneumoniae *proteins were 90, 97 and 90%, respectively.

**Table 1:** Proteins found by sequence and domain searches of the genome *C. jejuni *strain NCTC 11168 using *E. coli *enzymes involved in phosphonate utilisation

***E. coli *Protein**	***C. jejuni *Protein/Function**	**Sequence Similarity (%)**	**Domain Homology**
PhnH	Cj0641/Unknown function.	24	-----------
PhnK	Cj1663/Probable ATP-binding.	46	ABC_MJ0796
	Cj0774c/Probable binding protein.	50	ABC_MJ0796
PhnL	Cj1663/Probable ATP-binding.	49	ABC_MJ0796

Investigation of the genomic organisation of the genes *cj0641, cj0774c *and *cj1663 *indicated that they were not co-located, and none of the genes appeared to be co-transcribed with other genes.

### Isolation of *Campylobacter jejuni *genes

The three coding regions *cj0641, cj0774c *and *cj1663 *were amplified from *C. jejuni *strain 81116 and cloned into the vector pGEM-T Easy by standard cloning techniques. The primers used for the amplification of these genes given in Table 2 were designed based on the published nucleotide sequence of *C. jejuni *NCTC 11168 [17]. Sequence analyses of the genes cloned into the recombinant plasmids pGU0303 (pGEM-T EasyΩ*cj0641*), pGU0304 (pGEM-T EasyΩ*cj0774c*) and pGU0305 (pGEM-T EasyΩ*cj1663*) indicated that the amplified regions *cj0641, cj0774c *and *cj1663 *from *C. jejuni *81116, had a 96–99% nucleotide sequence identity with the sequences of the corresponding *C. jejuni *NCTC 11168 genes.

**Table 2:** Primers used to clone *C. jejuni *strain 81116 genes

**Name**	**Sequence**
*cj0641*-F	5'-CTT TTG CTT TGC TAA GAT TTG AT
*cj0641*-R	5'-TAA TCA TCA ATT TCC CCA GTC
*cj0774c*-F	5'-CTT CAT TCA TGA TGC CAC CTC C
*cj0774c*-R	5'-TGA ACT TCA AAA TCT AAG AGG T
*cj1663*-F	5'-GTG TGT GAA AAT TTG AAA GGT G
*cj1663*-R	5'-TCA TTT TAA CAC CCC ATG TTG
*cj1663*p2A INV	5'-GAA GAT CTC CCA TTG TCT AAG ATA TAC TCC C
*cj1663*p2B INV	5'-GAA GAT CTC CAA TAC TAT CAC TCA TGG ACA T

### In vitro mutagenesis of *Campylobacter jejuni *genes

Disruption of *cj0641 *in plasmid pGU0303 and of *cj0774c *in plasmid pGU0304 was performed by insertion of a non-polar *Km*^R ^cassette [18] within unique restriction sites *Cla*I and *Hind*III, respectively. No unique restriction sites existed in *cj1663 *which would serve to insert an antibiotic cassette in this gene. Hence, inverse PCR of pGU0305 was employed to generate *Bgl*II restriction sites for insertional inactivation of *cj1663*. The primers *cj1663*p2A INV and *cj1663*p2B INV were designed based on the nucleotide sequence of *cj1663 *cloned into recombinant plasmid pGU0305 (Table 1). Inverse PCR of pGU0305 generated a linearised plasmid with *Bgl*II sites incorporated into the primer termini, which was used to insert the *Km*^R ^cassette into *cj1663*. The insertion of the *Km*^R ^cassette within each gene was verified by restriction enzyme digest and sequence analysis.

The recombinant plasmids with disrupted *C. jejuni *genes pGU0306 (pGEM-T EasyΩ*cj064*1Ω*Km*^R^), pGU0307 (pGEM-T EasyΩ*cj0774c*Ω*Km*^R^) and pGU0308 (pGEM-T EasyΩ*cj1663*Ω*Km*^R^) were subsequently employed to generate isogenic mutants of *C. jejuni *81116.

### Generation and identification of C. jejuni 81116 isogenic mutants

The disrupted *cj0641, cj0774c *and *cj1663 *genes were introduced into the genome of wild-type *C. jejuni *by electroporating electro-competent *C. jejuni *81116 cells with plasmid DNA containing the mutated genes. Transformed bacteria could only be recovered on primary isolation plates.

**Figure 1:**
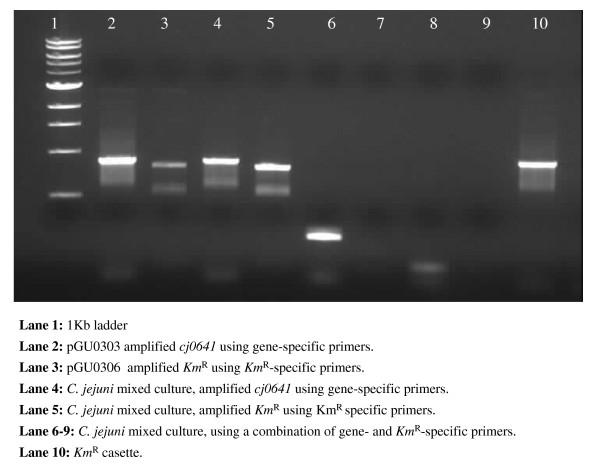
***Campylobacter jejuni *single colonies from the initial isolation plate**. Lane 1: 1 kb ladder. Lane 2: pGU0303 amplified *cj0641 *using gene-specific primers. Lane 3: pGU0306 amplified *Km*^R ^using *Km*^R^-specific primers. Lane 4: *C. jejuni *from the original isolation plate, amplified *cj0641 *using gene-specific primers. Lane 5: *C. jejuni *from the original isolation plate, amplified *Km*^R ^using *Km*^R^specific primers. Lane 6–9: *C. jejuni *from the original isolation plate, using a combination of gene- and *Km*^R^-specific primers. Lane 10: *Km*^R ^cassette.

To verify the presence of isogenic mutants, the transformants were initially grown on a TSA/HBA recovery plates without antibiotic selection. Subsequent plating onto kanamycin selective media resulted in cell death. All attempts to recover pure cultures of the isogenic mutants were unsuccessful. PCR analyses of bacterial DNA from single colonies on the initial recovery plates indicated that the genes disrupted by the insertion of the *Km*^R ^cassette were present in the genome of some of the cells, showing that the cultures which grew on the plates were a mixed bacterial cell population. Verification of the insertion of the kanamycin cassette was determined by amplification of the *Km*^*R *^cassette. Amplicons of either mutant or wild-type gene could not be recovered using the gene-specific primers from mixed populations (Figure 1).

Since none of the isogenic mutants could be isolated but grew in mixed cultures on primary recovery plates, it was hypothesised that the mutants may require a product or products produced by the wild-type strain to survive. Mixed cultures of wild-type and mutant bacteria harvested from primary isolation plates were grown in liquid "conditioned media" with or without 50 μg/ml kanamycin. In controls, mixed bacterial cultures were grown in liquid fresh media with or without kanamycin. In the presence of kanamycin *C. jejuni *81116 cells grew only in conditioned media (Table 3). The *C. jejuni *bacteria isolated from cultures grown in conditioned media with kanamycin were plated onto TSA/HBA plates with or without 50 μg/ml kanamycin, but no bacteria were recovered from any these cultures, suggesting that bacteria without the kanamycin cassette were screened out by the conditioned media with kanamycin.

**Table 3:** Growth rates of mixed cultures of *C. jejuni *strain 81116 wild-type and isogenic mutants in fresh and conditioned Brucella Broth with and without 50 μg/ml kanamycin.

**Growth media**	**Bacterial growth (cfu/ml)**			
	
	**wild-type**	**cj0641**^-^**+ wild-type**	**cj0774c^-^+ wild-type**	**cj1663^-^+ wild-type**
Brucella Broth	7.0 × 10^9^	6.0 × 10^9^	6.5 × 10^9^	7.0 × 10^9^
Brucella Broth + Kanamycin	-----	-----	-----	-----
Conditioned Brucella Broth	7.0 × 10^9^	8.5 × 10^9^	7.2 × 10^9^	7.8 × 10^9^
Conditioned Brucella Broth + Kanamycin	-----	2.0 × 10^8^	2.0 × 10^8^	4.4 × 10^8^

Cell growth was determined by measuring absorbances at 410 nm and converting these data to cfu/ml using a calibration curve

### Measurement of phosphonate degradation by *E. coli *lysates

Degradation of PhePhn, phosphonoacetate (PhnAce), AmePhn and PhePhi, was measured in whole-cell lysates of wild-type *E. coli *DH5α grown on LBA plates. The catabolism of phosphonate was confirmed as resulting from enzyme activities by heating the lysates for 4 h at 80°C, or by suspending them in 1% SDS and observing the abolition of catabolic activity.

**Figure 2:**
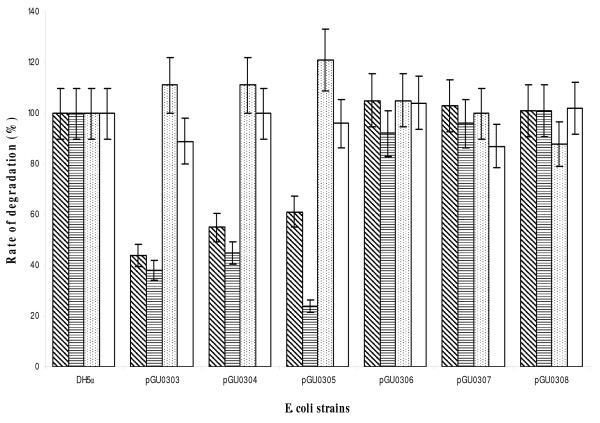
**Rates of degradation of four different substrates by *E. coli *strains**. Measurements were performed at 37°C, using one-dimensional ^1^H-NMR spectroscopy (n = 3). Control: wild-type; pGUO0303: mutant transformed with pGEM-T EasyΩ*cj0641*; pGUO0304: mutant transformed with pGEM-T EasyΩ*cj0774c*; pGUO0305: mutant transformed with pGEM-T EasyΩ*cj1663*; pGU0306: mutant transformed with pGEM-T EasyΩ*cj064*1Ω*Km*^R^; pGU0307: mutant transformed with pGEM-T EasyΩ*cj0774c*Ω*Km*^R^; and pGU0308: mutant transformed with pGEM-T EasyΩ*cj1663*Ω*Km*^R^. The activities of the wild-type were considered 100% and mutant activities are presented relative to the total control activity for the specific substrate. Histogram patterns correspond to degradation rates of *PhePhn*: inclined lines; *PhnAce*: horizontal lines; *AmePhn*: dots; and *PhePhi*: empty. The errors were calculated by determining the standard deviation from the mean of triplicate experiments.

Degradation of PhePhn, PhnAce and AmePhn was measured in *E. coli *whole-cell lysates of bacteria transformed with either of the plasmids pGEM-T Easy pGU0303, pGU0304, pGU0305, pGU0306, pGU0307 or pGU0308 (Figure 2). Similarly to untransformed wild-type bacteria, the lysates of cells transformed with pGU0303, pGU0304 and pGU0305 showed degradation of PhePhn and PhnAce, with significant reductions in the rates. AmePhn degradation was similar in cells transformed with pGU0303, pGU0304 and pGU0305 relative to the rates measured in the control. Transformation of *E. coli *with pGU0306, pGU0307 or pGU0308 had no effect on any of the three activities. In addition, no change was observed in the degradation of PhePhi between all strains (Figure 2).

## Discussion

Previous studies demonstrated that *Campylobacter *spp. catabolise various phoshonates [12], but sequence analyses of the *C. jejuni *strains 11168 and RM1221 genomes did not identify in this species orthologues of genes involved in phosphonate degradation in *E. coli *or *Salmonella typhimurium*. Nonetheless, the coding sequences *cj0641, cj0774c *and *cj1663 *showed some sequence or domain homology to genes encoding proteins of the C-P lyase of *E. coli*. These genes were disrupted in *C. jejuni *strain 81116 to determine whether the resultant mutants were altered in their ability to degrade phosphonates. The low sequence similarity between *E. coli *PhnH, PhnK and PhnL and *C. jejuni *proteins was in contrast with the high sequence similarity found with *Enterobacter sp. 638 *and *K. pneumoniae *proteins of the C-P lyase pathway, suggesting that the *C. jejuni *enzymes belong to different pathways.

The three genes from *C. jejuni *strain 81116 were cloned, and analyses of their sequences showed nucleotide identities of 96–99% with the *cj0641, cj0774c *and *cj1663 *genes of *C. jejuni *strain 11168. The data indicated that the genes were highly conserved, even more than the highly conserved *flaA *genes of *Campylobacter *spp. which have similarities between 73.6 and 92.3% [19]. The three genes were not co-located or co-transcribed in the *C. jejuni *11168 or 81116 genomes, and did not form part of operons.

The genes *cj0641, cj0774c *or *cj1663 *were inactivated by inserting a *Km*^R ^cassette, and *C. jejuni *81116 isogenic mutants were constructed by incorporation of each the disrupted genes into the genome of the wild-type strain through double allelic exchanges. The mutant bacteria were not viable, but were able to grow in mixed cultures with the wild-type strain. Growth experiments suggested that the mutants required for viability some factor(s) expressed by the wild-type strain. It is highly unlikely that a mutation in each of the three genes would result in identical phenotypes by chance. Inactivation of these genes by insertion of a *Km*^R ^cassette may have resulted in polar mutations. However, none of three genes belongs to a recognisable cluster or to an operon, and analyses of the genes downstream of *cj0641, cj0774c *and *cj1663 *indicated that they were not co-transcribed. In addition, the insertion of the *Km*^R ^cassette has been performed in numerous studies without having polar effects on downstream genes [18,20]. Thus, polar mutations probably were not the cause of the decreased survival. Alternatively, it is possible that these genes encode multifunctional proteins given that the *C. jejuni *genome is one of the most densely transcribed genomes known to date [17], and some of the functions of those proteins could be essential to the survival of *C. jejuni*.

The results of the bacterial cell culture experiments indicated the dependence of mutated *C. jejuni *on the wild-type strain for survival, suggesting that the mutants lacked the ability to produce, regulate, or utilise some compound(s) produced by the wild-type *C. jejuni *strain 81116.

Indirect evidence for the involvement of the proteins CJ0641, CJ0774c and CJ1663 in phosphonate degradation was obtained by expressing them in *E. coli *DH5α transformed with plasmids carrying each one of the genes. The C-P lyase enzyme of *E. coli *is expressed under conditions of phosphate starvation [6]. To ensure that this endogenous *E. coli *lyase was not expressed, bacteria were grown in the presence of inorganic phosphate. Hence, the catabolism of phosphonates observed in the transformed *E. coli *was the result of other enzyme activities (Figure 2).

Transformation of the wild-type *E. coli *strain with the plasmid pGEM-T Easy did not affect the rates of degradation of PhePhn, PhnAce and AmePhn. Transformation with a plasmid bearing *cj0641*, *cj0774c *or *cj1663 *altered the rate of PhePhn and PhnAce degradation relative to the wild-type (Figure 2). Transformation with a plasmid bearing one of the three genes inactivated with a kanamycin cassette had no effect on phosphonate degradation (Figure 2). The largest inhibition of PhePhn degradation was observed in *E. coli *transformed with *cj0641*, while the largest inhibition of PhnAce was due to transformation with *cj1663*. The relative decrease of phosphonate degradation between the three strains suggested that CJ0641 was more specific to PhePhn and CJ1663 was more specific to PhnAce. Comparison of the relative decrease of phosphonate degradation between substrates for each strain showed that *E. coli *transformed with pGU0303 and pGU0304 decreased PhnAce and PhePhn catabolism in equal amounts, while pGU0305 inhibited PhnAce degradation significantly more than that of PhePhn.

A reasonable expectation for the expression of an exogenous enzyme in a bacterium which also has a similar endogenous activity is that the overall activity measured in the transformed bacterium will be higher than in the parent organism. However, the measured activity is modulated by a number of factors including the relative abundance of both enzymes, their affinity for the substrate, their velocities and the substrate concentration. Regarding the latter, the overall measured activity will be higher when the concentration of the substrate is sufficient for both the endogenous and exogenous enzymes to operate as if the other were not present. Otherwise, both enzymes will compete for the substrate and depending on their relative affinities for the substrate and velocities, the overall measured activity may be higher, not significantly changed or lower. The latter case could occur when the exogenous enzyme has a much greater affinity and significantly lower velocity. In the case of PhePhn and PhnAce the substrate concentrations are well below saturation of the *C. jejuni *enzymes [12], hence the enzymes expressed in *E. coli *will compete with the endogenous activities, and the observed decrease of overall rates in the transformed bacteria could be explained by a competition of enzymes for the substrate

Several factors could be responsible for the lack of change in the rates of AmePhn degradation. *C. jejuni *has an enzyme system that degrades PhePhn and PhnAce and an independent system that degrades AmePhn, with the catabolism of AmePhn approximately half of that of the other two phosphonates [12]. This suggested that the former activities were due to different enzyme(s) than that of the latter. Also, the absence of changes in the rates of AmePhn degradation may be due to homologues of the broad specificity C-P lyase of *E. coli *[8] having different specificities in *C. jejuni*. Finally, the rate of AmePhn degradation may have undergone changes which were below the levels of detection owing to its significantly smaller value than the rate of PhePhn and PhnAce.

No change was observed in the degradation of PhePhi between all strains showing that the changes in activity are specific to phosphonate degradation, thus, supporting the interpretation that these genes are involved in phosphonate metabolism.

## Conclusion

The data showed that the gene products of *cj0641, cj0774c *and *cj1663 *were essential for the survival of the bacterium. In addition, evidence supported that CJ0641, CJ0774c and CJ1663 would be involved in phosphonate metabolism in the bacterium. The specific molecular events leading to changes in phosphonate degradation in transformed *E. coli *remain to be fully elucidated, but the study demonstrated that the *C. jejuni *proteins expressed in *E. coli *modulated the rates of phosphonate catabolism in the latter.

## Methods

### Materials

Brain Heart Infusion (BHI) Broth, Brucella Broth, Columbia Agar, Luria Broth, Tryptone Soya Agar (TSA) and defibrinated horse blood were from Oxoid (Heilderberg, VIC, Australia). Ampicillin and kanamycin, were purchased from Sigma (St Louis, MO, USA); and isopropyl-β-D-1-thioglactopyranoside (IPTG) and 5-bromo-4-chloro-3-indolyl-β-D-galactoside (X-Gal) were from Progen Biosciences (Archefield, QLD, Australia). Phosphonoacetic acid (PhnAce), α-aminomethylphosphonic acid (AmePhn), phenylphosphonic acid (PhePhn), and phenylphosphinic acid (PhePhi) were from Aldrich (Milwaukee, WI, USA). Sodium dodecyl sulfate (SDS) was from Amresco (Solon, OH, USA). All other reagents were of analytical grade.

### Bacterial strains, plasmids and growth conditions

The *C. jejuni *and *E. coli *strains, and the plasmids [21] used in this study are listed in Table 4. *Campylobacter jejuni *strains NCTC 11168 [22] and 81116 [23] were grown on Columbia Agar supplemented with 5% (v/v) defibrinated horse blood (HBA), or TSA supplemented with 5% (v/v) defibrinated horse blood and ampicillin or kanamycin when required. *C. jejuni *cells were incubated for 48 hours at 37°C under the microaerobic conditions 90% N_2_, 5% CO_2_, and 5% O_2_. Liquid cultures of *C. jejuni *were grown in Brucella Broth, at 37°C for 24–48 hours under the same microaerobic conditions as for plates. Cell growth was determined by measuring optical densities (OD) at 410 nm.

**Table 4:** Bacterial strains and plasmids used in the study

**Bacterial Strain or Plasmid**	**Origin and phenotype/Plasmid description**
*C. jejuni *11168	Human origin. Serotype O2. [22]
*C. jejuni *81116	Human origin, motile isolate. [23]
*E. coli *DH5α	*Sup*E44Ω*lac*U169 (80*lacZ*ΩM15) *hsdR*17 *rec*A1 *end*A1 *gyr*A96 *thi*-1 *rel*-A1 [21]
pGU0303	pGEM-T EasyΩ*cj0641 *(This study)
pGU0304	pGEM-T EasyΩ*cj0774c *(This study)
pGU0305	pGEM-T EasyΩ*cj1663 *(This study)
pGU0306	pGEM-T EasyΩ*cj0641*Ω*Km*^R ^(This study)
pGU0307	pGEM-T EasyΩ*cj0774c*Ω*Km*^R ^(This study)
pGU0308	pGEM-T EasyΩ*cj1663*Ω*Km*^R ^(This study)

"Conditioned" Brucella Broth to grow mutant *C. jejuni *bacteria was prepared by growing wild-type *C. jejuni *strain 81116 in Brucella Broth for 24 hours, removing the cells by centrifugation, and collecting and filtering the supernatant. The absence of viable cells in the supernatant was verified by plating it and observing no growth on the plates.

*Escherichia coli *strain DH5α was grown on Luria Broth Agar (LBA) plates supplemented with ampicillin, kanamycin, IPTG or X-Gal when required, and incubated at 37°C for 24 hours. Liquid cultures of *E. coli *were grown in Luria Broth with antibiotic supplements, ampicillin or kanamycin, where necessary, and under the same conditions as for plates.

### Genome searches

Sequences of *E. coli *K12 proteins [24] were employed to search in the fully annotated genomes of *C. jejuni *strains NCTC 11168 and RM1221, as well as the genomes of *Klebsiella pneumoniae *and *Enterobacter sp. 638 *for genes orthologous to those involved in C-P bond hydrolysis. The *E. coli *sequences were those encoded by the genes *phnG, phnH, phnI, phnJ, phnK, phnL*, and *phnM *(accession numbers P16685, P16686, P16687, P16688, P16678, P16679, and P16689) that are required for alkylphosphonate catalysis and likely constituting a membrane-associated carbon-phosphorus (C-P) lyase [2,7].

Sequence homology searches were performed using the BLASTP program for microbial genomes  of the National Center for Biotechnology Information (NCBI; Bethesda, MD, USA). Domain homology searches were carried out using the Conserved Domain Architecture Retrieval Tool (CDART) of NCBI .

### PCR amplifications

Primers for the genes *cj0641, cj0774c *and *cj1663 *in *C. jejuni *strain 81116, were designed using the published nucleotide sequence of *C. jejuni *strain NCTC 11168 [17]. Primer sequences are shown in Table 2. Protein similarity analyses comparing the sequenced genes of *C. jejuni *81116 to the published sequences of *C. jejuni *11168 were performed using BLASTP program for microbial genomes of the NCBI.

Standard PCR in 50 μl reaction mixtures were performed with the following parameters: 95°C for 4 minutes, 30 cycles of 94°C for 30 s, 52°C for 30 s, and 72°C for 2 min. Template DNA was prepared by the crude lysis boiling method, in which a single *C. jejuni *colony was boiled for 5 min in 150 μl of water and cellular debris were removed by centrifugation.

### Cloning and screening of recombinant plasmids pGU0303, pGU0304 and pGU0305

Amplified PCR fragments were cloned into the bacterial cloning vector system pGEM-T Easy (Promega; Madison, WI, USA) in *E. coli *by standard cloning techniques [25]. The plasmids pGU0303, pGU0304 and pGU0305 each containing one of the three *C. jejuni *genes are described in Table 4. Transformant colonies putatively carrying recombinant plasmids were screened by blue/white colony selection, and further verified by PCR, using plasmid specific primers for the T7 and SP6 polymerase promoters, which flank the multiple cloning region of pGEM-T Easy. Prior to mutagenesis, constructs were sequenced by di-dioxynucleotide sequencing, to verify the correct base pair order.

In vitro mutagenesis of cloned Campylobacter jejuni genes cj0641, cj0774c and cj1663 Mutagenesis was performed by insertional inactivation of the genes *cj0641*, *cj0774c *and *cj1663 *using a non-polar kanamycin antibiotic resistance cassette *Km*^R ^[26,27]. The *Km*^R ^cassette was amplified from the plasmid pMW2 using primers with *Bgl*II sites incorporated into the termini (Table 2). The *Km*^R ^cassette was inserted into unique restriction sites within the cloned *C. jejuni *inserts. Linearised plasmids for pGU0303 (pGEM-T EasyΩ*cj0641*), and pGU0304 (pGEM-T EasyΩ*cj0774c*) were generated by restriction enzyme digestion with *Cla*I and *Hind*III respectively, according to the manufacturer's instructions.

The plasmid pGU0305 (pGEM-T EasyΩ*cj1663*) was linearised by inverse PCR, as no unique restriction sites existed within *cj1663*. The primers *cj1663*p2A INV and *cj1663*p2B INV were designed based on the nucleotide sequence of pGU0305 and created a small deletion within *cj1663*. Inverse PCR in 50 μL reaction mixtures was conducted as follows: 95°C for 5 min, 40 cycles 94°C for 30 s, 52°C for 1 min, and 72°C for 5 min. The Eppendorf TripleMaster *Taq *was the DNA polymerase used for inverse PCR (Eppendorf; North Ryde, NSW, Australia).

### *Campylobacter jejuni *isogenic mutants

Electrocompetent *C. jejuni *strain 81116 cells were prepared [28] and transformed with mutated recombinant plasmids using a BioRad MicroPulser (BioRad; Regents Park, NSW, Australia) at a voltage of 2.48 V, and time constant of 5 min. The contents of the 2 mm cuvettes were flushed with BHI broth and allowed to recover at 37°C for 5 h under microaerobic conditions. Reactions were plated onto TSA/HBA plates.

### Measurement of phosphonate catabolism using nuclear magnetic resonance spectroscopy

Wild-type and transformed *E. coli *strain DH5α cells were grown on LBA plates supplemented with 50 μg/ml ampicillin and incubated at 37°C for 6 h. The bacteria were harvested in 150 mM sodium chloride (NaCl) and centrifuged 16,000 × *g *at 4°C for 10 min. The pellet was collected and the supernatant discarded. The pellet was resuspended in 14 ml 150 mM NaCl solution and washed twice more. Cells were lysed by twice freezing in liquid nitrogen and thawing.

Catabolism of AmePhn, PhePhn, PhePhi and PhnAce was measured employing proton nuclear magnetic resonance (^1^H-NMR) spectroscopy. Lysates (150 μl) were suspended in 150 mM NaCl and mixed with ^2^H_2_O (50 μl), 150 mM KCl (50 μl), and 150 mM NaCl (150 μl) before phosphonate was added at time zero to give a final volume of 600 μl. Initial concentrations of phosphonate substrates were 120 mM. Suspensions of bacterial lysates were placed in 5 mm NMR tubes (Wilmad; Buena, NJ, USA) and measurements of enzyme activities were carried out at 37°C. ^1^H-NMR free induction decays were collected using a Bruker DMX-600 spectrometer, operating in the pulsed Fourier transform mode with quadrature detection. The instrumental parameters for the spectrometer were: operating frequency 600.13 MHz, spectral width 6009.61 Hz, memory size 16 K, acquisition time 3.61 s, number of transients 64, pulse angle 50° (*ca. *3 μs) and relaxation delay with solvent presaturation 1.7 s. Spectral resolution was enhanced by Gaussian multiplication with line broadening of 0.7 Hz and Gaussian broadening factor of 0.19. Proton spectra were acquired with presaturation of the water resonance. The time-evolution of substrates and products were followed by acquiring sequential spectra of the reactions. Enzyme rates were obtained by measuring the decrease over time of the intensity of resonances arising from the phosphonate compounds. Calibrations of substrate peaks were performed by extrapolating the resonance intensity data to zero time and assigning to this intensity the original concentration value.

## Competing interests

The authors declare that they have no competing interests.

## Authors' contributions

LH carried out the molecular genetic studies and contributed to draft the manuscript. NOK carried out the enzyme assays and contributed to draft the manuscript. JLF assisted in the enzyme assays and corrections to the manuscript. VK participated in the design and coordination of the molecular genetic studies, and contributed to draft the manuscript. GLM conceived and coordinated the study, participated in its design, the interpretation of results, and helped to draft the manuscript.
